# Thymosin Beta-4 and Ciprofloxacin Adjunctive Therapy Improves *Pseudomonas aeruginosa*-Induced Keratitis

**DOI:** 10.3390/cells7100145

**Published:** 2018-09-20

**Authors:** Thomas W Carion, Abdul Shukkur Ebrahim, David Kracht, Aditya Agrawal, Eliisa Strand, Omar Kaddurah, Cody R. McWhirter, Gabriel Sosne, Elizabeth A. Berger

**Affiliations:** Department of Ophthalmology, Visual & Anatomical Sciences, Wayne State University School of Medicine, Detroit, MI 48201, USA; tcarion@med.wayne.edu (T.W.C.); eabdulsh@med.wayne.edu (A.S.E.); dkracht14@gmail.com (D.K.); aditya.agrawal@med.wayne.edu (A.A.); eliisa.strand@wayne.edu (E.S.); okaddura@med.wayne.edu (O.K.); cmcwhirt@med.wayne.edu (C.R.M.); gsosne@med.wayne.edu (G.S.)

**Keywords:** corneal infection, wound healing, pathogenesis, bacteria, immunoregulation

## Abstract

With increasing multidrug resistance and contraindication for corticosteroid use, the goal of this study was to develop thymosin beta-4 (Tβ4) as an adjunctive therapy to antibiotics for the treatment of bacterial keratitis that effectively promotes enhanced wound healing, host defense, and inflammation resolution. Disease outcome was assessed by clinical score, slit lamp photography, and histopathology. Cytokine profile, bacterial load, PMN infiltration, and Griess and reactive oxygen species (ROS) levels were determined. Adjunct Tβ4 treatment resulted in a significant improvement compared to PBS, Tβ4, and most remarkably, ciprofloxacin, correlating with changes in mediators of inflammation and wound healing. Collectively, these data provide evidence that wound healing is an essential aspect in the development of new therapies to treat corneal infection. Use of adjunctive Tβ4 provides a more efficacious approach for bacterial keratitis by addressing both the infectious pathogen and deleterious host response.

## 1. Background

Microbial keratitis is a rapidly progressing, visually debilitating infection of the cornea that can lead to corneal scarring, endophthalmitis, and perforation. Corneal opacification, a complication of keratitis, is among the leading causes of legal blindness worldwide, second to cataracts. *Pseudomonas aeruginosa* and *Staphylococcus aureus* are the two bacteria most commonly associated with this type of infection [1,2,3,4]. In addition, reports indicate that up to 38% of fungal keratitis cases are co-infected with bacteria [5]. Risk factors include patients who are immunocompromised, those who have undergone refractive corneal surgery, and those with prior penetrating keratoplasty, as well as extended wear contact lens users [6]. Current treatment of microbial keratitis primarily addresses the pathogen using antibiotics. Bacterial clearance is of utmost importance, yet does not guarantee good visual outcome. Fortified antibiotic use has been shown to be toxic to the corneal epithelium and inhibit the healing process necessary for restoring visual acuity [7,8]. As resistance to antibiotics gains prevalence, microbial keratitis has become an important global healthcare issue [1,2]. Clinicians are often left to rely upon the eye’s innate ability to heal itself, as there are limited options beyond antibiotics and corticosteroids for treating patients with corneal infection. Beyond antibiotics, agents in use, such as lubricating ointments, artificial tears, and anti-inflammatory drops, do not fully accommodate clinical needs and have many harmful complications. In particular, topical corticosteroids are often used to reduce the host inflammatory response. Yet, controversy remains regarding the benefits and risks associated with this immunosuppressive therapy [9,10]. To this end, treatments are needed that both regulate the inflammatory response and promote corneal wound healing to resolve visual disturbances and improve quality of life. 

Tβ4 is a small, naturally occurring 43-amino-acid protein that promotes wound healing and reduces corneal inflammation [11]. It is highly conserved across species and is expressed in all tissues and cell types except red blood cells [12]. Initially, Tβ4 was thought to act solely as an actin-sequestering molecule, but is now recognized for its importance in wound healing—a therapeutic aspect that is severely lacking in current treatment options for the eye. In fact, several regenerative properties have been attributed to Tβ4, including: full thickness dermal wound repair [13,14], stem cell recruitment and differentiation [11], modulation of wound site inflammation [15], antiapoptotic effects [16,17], and antimicrobial activity [18]. Three phase II clinical trials for dry eye syndrome report no adverse effects with Tβ4 treatment [19,20,21]. The current study investigates how Tβ4 improves disease pathogenesis associated with ocular infection. Our findings suggest that topical adjunctive Tβ4 treatment can elicit the desired therapeutic response, including reduction in corneal inflammation and rapid corneal re-epithelialization. Adjunctive Tβ4 treatment holds novel therapeutic potential to regulate and, optimally, resolve disease pathogenesis in the cornea and perhaps other infectious and immune-based inflammatory disease. We establish the importance of Tβ4 as a therapeutic agent in conjunction with antibiotics with high impact for immediate clinical development.

## 2. Methods

### 2.1. Experimental Animal Protocol

The left central cornea of 8-week-old C57BL6 (B6) female mice (The Jackson Laboratory, Bar Harbor, ME, USA) were scarified as previously described [22]. A 5 µL aliquot containing 10^6^ CFU/mL of the cytotoxic *Pseudomonas aeruginosa* strain ATCC 19660 was applied topically to the wounded corneal surface. Mice were randomized into four different treatment groups consisting of either PBS as controls, Tβ4 (0.1%), ciprofloxacin (0.3%), or Tβ4 + ciprofloxacin, administered topically (5 μL) 3× per day beginning 24 h after infection. Uninfected, normal corneas were appropriately used as an additional control where noted. All animals were treated in a manner authorized by Wayne State University Institutional Animal Care and Use committee (protocol 16-090) and conformed to the Association for Research in Vision and Ophthalmology’s statement on the Use of Animals in Ophthalmic and Vision Research.

### 2.2. Ocular Response to Bacterial Infection

Infected eyes were observed daily in a blinded fashion and disease response was graded using an established grading scale [23] for statistical comparison. Mice were examined and a clinical score was calculated for 3 and 5 days postinfection (p.i.) for each group to express disease severity as mean clinical score ± SEM. Slit-lamp photography was used to illustrate disease response at corresponding time points. 

### 2.3. Histopathology

Whole eyes were enucleated from each treatment group at 5 days p.i. and processed for histopathological analysis. Briefly, eyes were immersed in PBS, rinsed, and fixed in 1% osmium tetroxide, 2.5% glutaraldehyde, and 0.2 M Sorenson’s phosphate buffer (pH 7.4) at a 1:1:1 ratio for 3 h at 4 °C. Eyes were rinsed (0.1 M phosphate buffer), dehydrated in graded ethanols and propylene oxide, and next infiltrated and embedded in Epon-araldite. Sections (1.5 μm thick) were stained with Richardson’s stain, observed, and photographed (Leica DM4000B, Leica Microsystems, Inc., Wetzlar, Germany).

### 2.4. Bacterial Load

Corneas from B6 animals treated with PBS, Tβ4, ciprofloxacin, or Tβ4 + ciprofloxacin were collected at 5 days p.i. and the number of viable bacteria was quantitated. Individual corneas were homogenized in sterile 0.9% saline containing 0.25% BSA. Serial 10-fold dilutions of each sample were plated on *Pseudomonas* isolation agar in triplicate and plates were incubated overnight at 37 °C. Results are reported as CFU/cornea ± SEM.

### 2.5. Measurement of ROS Levels

At 5 days p.i., corneal lysates from each treatment group were incubated in reaction buffer (130 mM KCl, 5 mM MgCl_2_, 20 mM NaH_2_PO_4_, 20 mM Tris-HCL, pH 7.4, 30 mM D-glucose, 7.5 μM DCFH-DA; ThermoFisher Scientific, Waltham, MA, USA) for 1 h at 37 °C [24]. Negative controls were incubated in reaction buffer without DCFH-DA. Formation of dichlorofluorescein (DCF), a detectable fluorescent product of oxidized DCFH-DA [25], was measured (SpectraMax M3 Multi-Mode reader; Molecular Devices, Sunnyvale, CA, USA). The final fluorescent values were acquired by calculating the DCF minus negative control values. Results are reported as fluorescence intensity ± SD.

### 2.6. Myeloperoxidase (MPO) Assay

An MPO assay was performed to correlate the number of PMN in infected corneas of B6 mice treated with PBS, Tβ4, ciprofloxacin, or Tβ4 + ciprofloxacin. Corneas were removed at 5 days p.i. and homogenized in 1.0 mL of 50 mM phosphate buffer (pH 6.0) containing 0.5% hexadecyltrimethyl-ammonium (HTAB). Samples were processed as previously described [26]. The change in absorbency (460 nm) was monitored for 5 min at 30 s intervals. The slope of the line was determined for each sample and used to calculate units of MPO/cornea. One unit of MPO activity is equivalent to ~2 × 10^5^ PMN [27] with a sensitivity of 0.90 units. 

### 2.7. Greiss Reaction

NO levels were determined (as previously described [28]) by measurement of its stable end product, nitrite, using a Greiss reagent (1% sulfanilamide/0.1% naphthylethylene diamine dihydrochloride 12.5% H3PO4) in corneas of PBS-, Tβ4-, ciprofloxacin-, and Tβ4 + ciprofloxacin-treated B6 mice. Corneas were processed as previously described [26]. Absorbance (570 nm) was measured and nitrite concentrations were estimated using a standard curve of sodium nitrite. Data are represented as the mean μM nitrite/cornea ± SD.

### 2.8. Real-Time RT-PCR

Total RNA was isolated from individual whole corneas at 3 and 5 days p.i. for gene expression analysis using RNA-STAT 60 (Tel-Test, Friendswood, TX, USA) according to the manufacturer’s recommendations and quantified by spectrophotometric determination (260 nm). 100 ng of total RNA was reverse transcribed and used to produce a cDNA template and amplified as previously described [29]. All primers for the PCR reaction were designed using Primer3 (Table 1). Semiquantitative real-time RT-PCR was performed using the CFX Connect Real-Time RT-PCR Detection System (BioRad, Hercules, CA, USA). PCR amplification conditions were determined using routine methods [30]. Relative transcript levels were determined using the relative standard curve method comparing the amount of target normalized to an endogenous reference, β-actin. Data are shown as the mean ± SD for relative transcript levels normalized to β-actin and relative to the expression of uninfected (normal) controls.

### 2.9. ELISA Analysis

Normal (uninfected) and infected corneas were removed at 3 and 5 days p.i. from B6 mice treated with PBS, Tβ4, ciprofloxacin, or Tβ4 + ciprofloxacin. Individual corneas were homogenized in 1 mL RIPA buffer (Cell Signaling Technology, Danvers, MA, USA) with a protease inhibitor cocktail (Thermo Fisher Scientific, Waltham, MA, USA). An aliquot of each supernatant was assayed in duplicate for IL-1β and MIP-2 per the manufacturer’s instruction (R&D Systems, St. Paul, MN, USA). The reported sensitivity of these assays was 4.8 pg/mL for IL-1β and 1.5 pg/mL for MIP-2. Results are expressed as average ng of each cytokine/mL ± SEM.

### 2.10. Statistical Analysis

Sample sizes were determined statistically prior to experimentation based on previous work [26,28,29,31], which includes a <5% mortality rate. Using a power analysis, we assume a mean difference = 2, standard deviation = 1, α = 0.05, power = 0.8, and a sample size ratio = 1. The difference in clinical scores between two groups at each time point was tested by the Mann–Whitney *U* test (GraphPad Prism; GraphPad Software, La Jolla, CA, USA). For all other experiments, a one-way ANOVA followed by Bonferroni’s multiple comparison test (GraphPad Prism) was used for analysis of 3+ groups. An unpaired Student’s *t* test was used for comparison between 2 groups. Data were considered significant at *P* < 0.05. A minimum of *N* = 5/group/time point were used unless otherwise stated. All experiments and/or measurements were carried out in triplicate and representative data from a typical experiment is shown.

## 3. Results

### 3.1. Tβ4 Adjunct Therapy Significantly Improves Disease Response 

**Figure 1:**
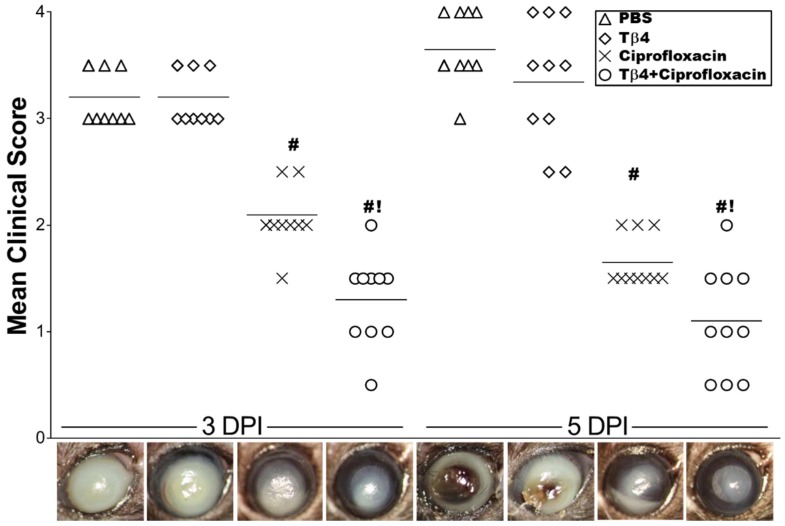
Ocular disease response of *P. aeruginosa*-infected B6 mice. Corneal response was graded at 3 & 5 days p.i. (*N* = 10 mice/group). Results are represented as mean clinical scores ± SEM. Photographs taken by slit-lamp provide further visualization of the differences in clinical scores between the different treatment groups and include: PBS (∆), Tβ4 (◊), ciprofloxacin (×), and Tβ4 + ciprofloxacin (○). ^#^ *P* < 0.05 versus PBS; ^!^ *P* < 0.05 versus ciprofloxacin.

Clinical scores, accompanied by representative photographs, were recorded (Figure 1) to initially assess the disease response. Tβ4 treatment was similar to PBS controls, ranging from dense opacity covering the entire anterior segment (+3) at 3 days to corneal melting and perforation (+4) by 5 days p.i. Disease response was significantly improved in ciprofloxacin-treated mice, demonstrating dense opacity, partially or fully covering the pupil (+2) at 3 days with increasing progress at 5 days p.i. Infected corneas treated with Tβ4 + ciprofloxacin exhibited the most improvement in disease severity (+1) with only moderate opacity covering the pupil at 3 days p.i., which was further reduced to slight central opacity at 5 days and significantly improved over all three treatment groups.

**Figure 2:**
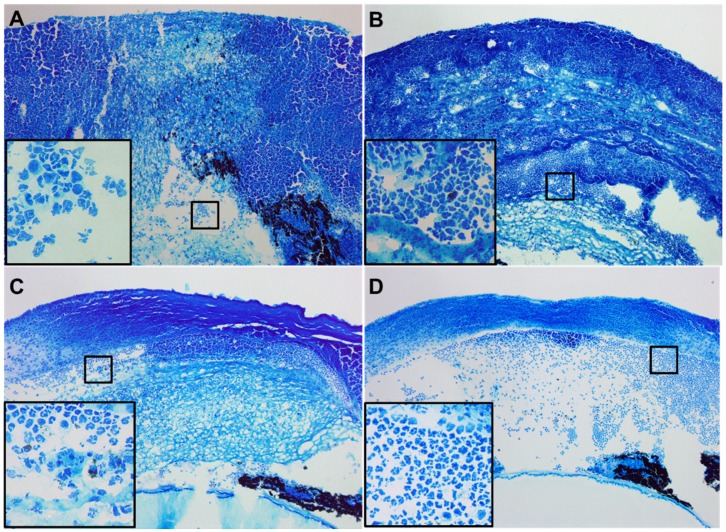
Histopathology at 5 days p.i. following PBS (**A**), Tβ4 (**B**), ciprofloxacin (**C**), and Tβ4 + ciprofloxacin (**D**) treatment. Whole eyes were sectioned (1.5 μm thick) and stained with Richardson’s stain. *N* = 5 corneas /group. Magnification = ×10; (×40, insets).

Histopathological sections from each treatment group (Figure 2) further confirm the observed disease response. Staining of PBS-treated controls (Figure 2A) revealed a completely denuded epithelium, massive edema, and excessive stromal degradation accompanied by heavy cellular infiltrate throughout the anterior chamber. Tβ4 treatment (Figure 2B) appeared very slightly improved with a somewhat less stromal degradation comparatively, but still edematous with a heavy cellular infiltrate throughout the stroma and anterior chamber. While the bacteria appeared to be cleared, ciprofloxacin treatment (Figure 2C) exhibited stromal edema, infiltrating cells within the anterior chamber and a disrupted, detached epithelium. The combination treatment (Figure 2D) revealed little to no edema with markedly reduced inflammatory cell infiltrate and a mostly intact epithelium indicative of activated wound healing.

### 3.2. Tβ4 Adjunct Treatment Significantly Reduces Bacterial Load, ROS, MPO, and Nitrite Levels

**Figure 3:**
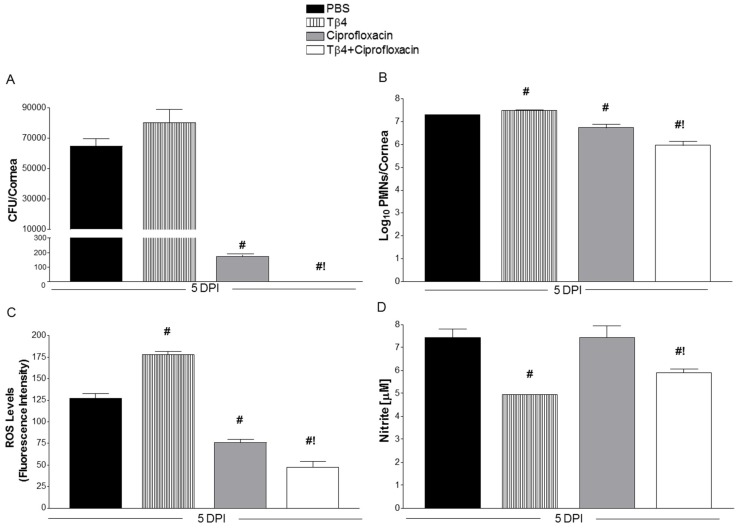
(**A**) Quantitation of viable bacteria detected at 5 days p.i. from corneas of PBS-, Tβ4-, ciprofloxacin-, and Tβ4 + ciprofloxacin-treated animals. Results are reported as CFU/cornea ± SD. (**B**) PMN numbers as correlated from levels of MPO detected from corneal lysates after infection. Results are reported as log_10_ PMNs/cornea ± SD. (**C**), ROS levels determined by fluorescent values (DCFH-DA values—negative control values). Fluorescent values were acquired by subtraction of negative control values from DCFH-DA values. Results are reported as mean intensity ± SD. (**D**) Nitrite levels as measured from corneal lysates after infection. Results are reported as μM nitrite/cornea ± SD. *N* = 5 corneas/group/time point. ^#^ *P* < 0.05 versus PBS; ^!^ *P* < 0.05 versus ciprofloxacin.

Viable bacteria were determined by direct plate counts from infected corneas at 5 days p.i., as shown in Figure 3A. No differences were detected between PBS and Tβ4 treatment groups. As expected, ciprofloxacin significantly reduced the bacterial burden by ~350-fold. However, Tβ4 + ciprofloxacin significantly decreased the bacterial load even further compared to all three treatment groups and, in fact, was undetectable. MPO assay (Figure 3B) revealed that PMN infiltration into infected corneas was higher in Tβ4 versus PBS treatment groups at 5 days. In contrast, MPO activity was significantly decreased with ciprofloxacin treatment, while Tβ4 + ciprofloxacin treatment resulted in further reduction in MPO levels compared to all three treatment groups, suggesting that the combination treatment had the strongest influence on reducing PMN infiltration into the infected corneas. Levels of ROS (Figure 3C), key signaling molecules known to play a major role during an inflammatory response, were significantly elevated in Tβ4- compared to PBS-treated corneas at 5 days after infection, but significantly decreased following ciprofloxacin treatment. Again, Tβ4 + ciprofloxacin further reduced ROS levels, as detected by DCFH-DA. Nitric oxide (NO) is a reactive free radical produced predominately by macrophages during inflammation. To correlate macrophage activation in response to infection, nitrite (a stable oxidized product of NO) was measured at 5 days p.i. for each treatment group (Figure 3D). Corneas of ciprofloxacin-treated mice showed similar nitrite levels when compared to PBS controls. Only Tβ4 and Tβ4 + ciprofloxacin treatment groups resulted in significantly reduced nitrite levels following infection.

### 3.3. Tβ4 Adjunct Treatment Modulates Key Proinflammatory Mediators

**Figure 4:**
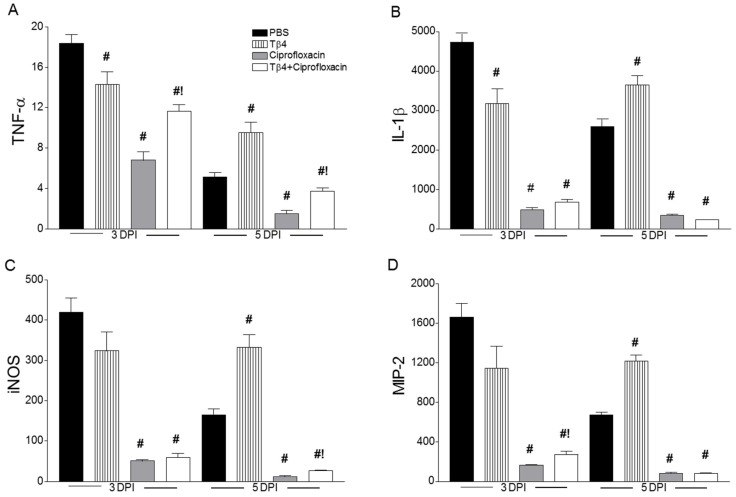
mRNA levels of select cytokine and chemokines at 3 and 5 days p.i. in corneas of B6 mice after PBS, Tβ4, ciprofloxacin, and Tβ4 + ciprofloxacin treatment. mRNA expression levels for TNF-α (**A**), IL-1β (**B**), iNOS (**C**), and MIP-2 (**D**) are normalized to β-actin and represented as relative fold change for the gene of interest compared to normal (uninfected) controls ± SEM. *N* = 8 corneas/group/time point. ^#^ *P* < 0.05 versus PBS; ^!^ *P* < 0.05 versus ciprofloxacin.

**Figure 5:**
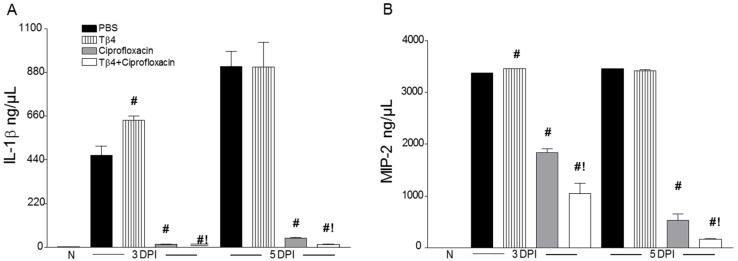
Protein levels of IL-1β (**A**) and MIP-2 (**B**) in corneas of PBS-, Tβ4-, ciprofloxacin-, and Tβ4 + ciprofloxacin-treated B6 mice at 3 and 5 days p.i. and normal, uninfected controls. Results are reported as mean (ng/mL) ± SEM. *N* = 5 corneas/group/time point. ^#^ *P* < 0.05 versus PBS; ^!^ *P* < 0.05 versus ciprofloxacin.

Select proinflammatory mediators, known to play a role in bacterial keratitis [26], were investigated to determine the immunoregulatory influence of Tβ4. As presented in Figure 4, transcript levels of TNF-α (Figure 4A), IL-1β (Figure 4B), iNOS (Figure 4C), and MIP-2 (Figure 4D) were analyzed in whole corneal lysates of the four treatment groups at 3 and 5 days p.i. Results indicated that corneal mRNA expression of proinflammatory cytokines/chemokines were all upregulated in PBS-treated animals at both time points. Compared to controls, Tβ4 treatment lowered these same gene transcripts at 3 days p.i., but then comparatively increased at 5 days. Ciprofloxacin and Tβ4 + ciprofloxacin treatment significantly reduced levels of proinflammatory mediators. As shown in Figure 5, when selectively confirmed at the protein level, IL-1β (Figure 5A) and MIP-2 (Figure 5B) followed similar trends, where PBS and Tβ4 treatment groups exhibited sustained production of both molecules over time. Ciprofloxacin significantly decreased IL-1β and MIP-2, but levels were even further reduced with the Tβ4 combination treatment. Overall, it appears that improved disease outcome subsequent to the adjunct treatment is due, in part, to a decreased proinflammatory cytokine/chemokine profile.

### 3.4. Treatment Activates Corneal Wound Healing Pathways after Infection

**Figure 6:**
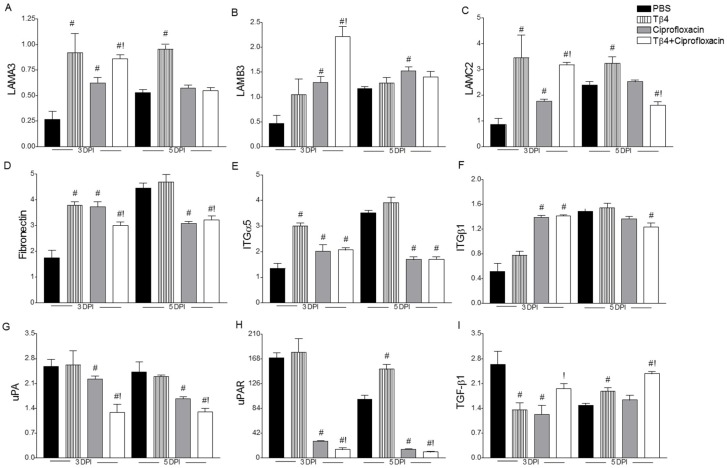
Transcript levels of select wound healing genes in corneas of PBS-, Tβ4-, ciprofloxacin-, and Tβ4 + ciprofloxacin-treated B6 mice at 3 and 5 days p.i. mRNA expression levels for LAMA3 (**A**), LAMB3 (**B**), LAMC2 (**C**), fibronectin (**D**), ITGα5 (**E**), ITGβ1 (**F**), uPA (**G**), uPAR (**H**), and TGF-β1 (**I**) are normalized to β-actin and represented as relative fold change for the gene of interest compared to normal (uninfected) controls ± SEM. *N* = 8 corneas/group/time point. ^#^ *P* < 0.05 versus PBS; ^!^ *P* < 0.05 versus ciprofloxacin.

mRNA levels of laminin α3 (LAMA3), laminin β3 (LAMB3), laminin γ2 (LAMC2), fibronectin, integrin α5 (ITGα5), ITGβ1, urokinase (uPA), urokinase receptor (uPAR), and TGF-β1 were measured at 3 and 5 days following infection in the four treatment groups, as shown in Figure 6A–I, respectively. The three subunits, α/β/γ, were examined given the prominent role of laminins as extracellular matrix glycoproteins required for cell adhesion and migration. α3 and γ2 chains were significantly increased in both Tβ4 treatment groups at 3 days p.i. compared to PBS and ciprofloxacin; levels remained elevated at 5 days in the Tβ4 group only. Increased expression of β3 was limited to the adjunctive treatment at 3 days p.i. only. Given previous work regarding Tβ4 and laminin-332, we next focused on other components of wound healing. Fibronectin mRNA levels were up early in Tβ4-, ciprofloxacin-, and Tβ4 + ciprofloxacin-treated animals versus PBS, then the latter two groups became significantly decreased at 5 days compared to both PBS and Tβ4 treatments. This same trend was observed for ITGα5 and ITGβ1. ITGα5 binds to the major binding site of fibronectin, while ITGβ1 interacts with the minor binding site, further promoting cell adhesion. While the fibronectin:integrin interaction plays a central role in corneal epithelial cell wound healing and signals the cells to migrate, detachment of epithelial cells from the fibronectin extracellular matrix is also required for active movement. In this regard, we next looked at uPA and uPAR, which promote matrix remodeling and cell activation, and TGF-β1, a regulator of the uPA/uPAR pathway [32]. Both uPA and uPAR levels were significantly decreased in ciprofloxacin-treated animals and further reduced with the combination treatment during infection when compared to PBS. However, only the Tβ4 and combination treatments enhanced expression of the anti-inflammatory molecule, TGF-β1, at 5 days p.i. versus PBS, while the combination treatment significantly increased TGF-β1 at both time points compared to ciprofloxacin alone.

**Figure 7:**
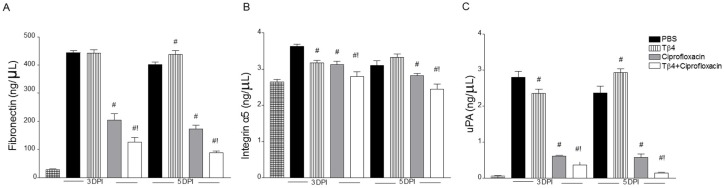
Protein levels of fibronectin (**A**), ITGα5 (**B**), and uPA (**C**) in corneas of PBS-, Tβ4-, ciprofloxacin-, and Tβ4 + ciprofloxacin-treated B6 mice at 3 and 5 days p.i. and uninfected (normal) controls. Results are reported as mean (ng/mL) ± SEM. *N* = 5 corneas/group/time point. ^#^ *P* < 0.05 versus PBS; ^!^ *P* < 0.05 versus ciprofloxacin.

Protein levels of fibronectin (Figure 7A), ITGα5 (Figure 7B), and uPA (Figure 7C) were selectively confirmed by ELISA at 3 and 5 days after infection, as shown in Figure 7. Results showed that all three molecules were significantly reduced in corneas of Tβ4 + ciprofloxacin-treated mice compared to all other treatment groups. Ciprofloxacin treatment was similarly reduced compared to PBS and Tβ4 alone, but remained elevated compared to the adjunct therapy.

### 3.5. Tβ4 Influences a Lipid Mediator Circuit Known to Play a Role in Epithelial Wound Healing

**Figure 8:**
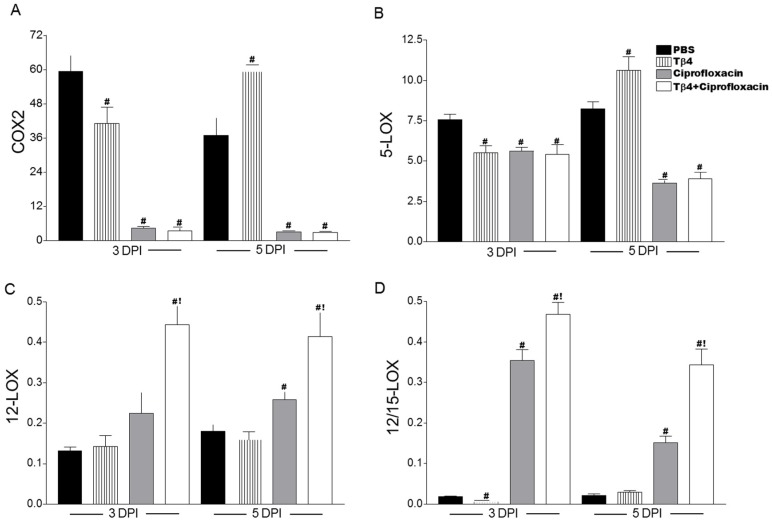
Real-time RT-PCR of lipid mediator biosynthetic enzymes, COX-2 (**A**), 5-LOX (**B**), 12-LOX (**C**), and 12/15-LOX (**D**) in corneas of PBS-, Tβ4-, ciprofloxacin-, and Tβ4 + ciprofloxacin-treated B6 mice at 3 and 5 days p.i. mRNA expression levels are normalized to β-actin and represented as relative fold change for the gene of interest compared to normal (uninfected) controls ± SEM. *N* = 8 corneas/group/time point. ^#^ *P* < 0.05 versus PBS; ^!^ *P* < 0.05 versus ciprofloxacin.

To begin elucidating potential mechanisms by which Tβ4 exerts its proresolving effects on the inflammatory response, we assessed expression of lipid mediator biosynthetic cyclooxygenase (COX) and lipoxygenase (LOX) enzymes, as shown in Figure 8. COX-2 (Figure 8A), an enzyme that is rapidly induced during inflammation, and 5-LOX (Figure 8B), an enzyme regarded as a marker of inflammatory leukocytes, were significantly decreased after infection in both ciprofloxacin and Tβ4 + ciprofloxacin treatment groups. In contrast, 12-LOX (Figure 8C), an enzyme associated with the production of proresolving lipid mediators, and 12/15-LOX (Figure 8D), a key marker of activated epithelial and mucosal proresolving pathways, were significantly enhanced in the Tβ4 + ciprofloxacin-treated animals compared to all other treatment groups; however, 12-LOX and 12/15-LOX were significantly increased compared to PBS controls after ciprofloxacin treatment at 3 days and 3 and 5 days p.i., respectively. 

**Figure 9:**
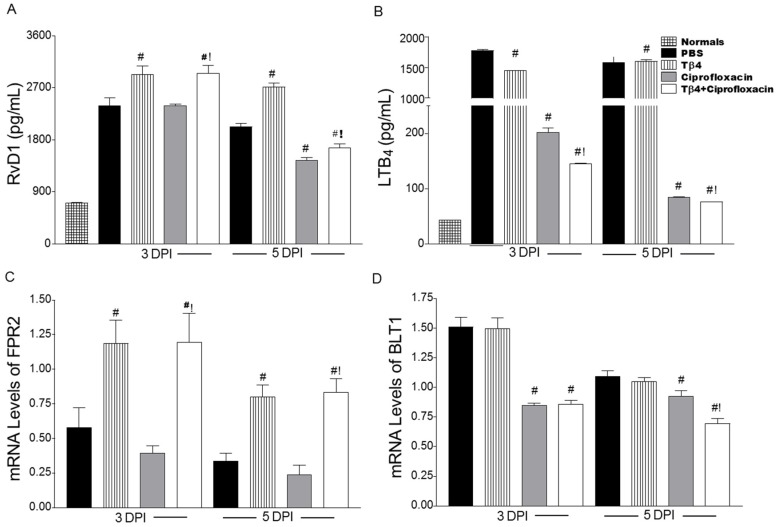
Quantification of RvD1 (**A**) and LTB4 (**B**) and mRNA expression levels for FPR2 (**C**) and BLT1 (**D**) as detected in corneas of PBS-, Tβ4-, ciprofloxacin-, and Tβ4 + ciprofloxacin-treated B6 mice at 3 and 5 days p.i. and normal, uninfected controls (protein analysis only). Protein results are reported as mean (pg/mL) ± SEM. *N* = 5 corneas/group/time point. mRNA expression levels are represented as relative fold change for the gene of interest compared to β-actin ± SEM. *N* = 8 corneas/group/time point. ^#^ *P* < 0.05 versus PBS; ^!^ *P* < 0.05 versus ciprofloxacin.

We next examined select lipid mediator end products, proresolving RvD1 and proinflammatory LTB4, along with their respective receptors, FPR2 and BLT1. As shown in Figure 9, RvD1 (Figure 9A) protein levels were significantly enhanced in both Tβ4 treatment groups compared to PBS and ciprofloxacin alone at both time points following infection. Following this trend, mRNA levels of the RvD1 receptor, FPR2 (Figure 9C), were significantly enhanced at both time points after Tβ4 treatment—both alone and with ciprofloxacin. Consistent with a reduced inflammatory response, Tβ4 + ciprofloxacin-treated mice revealed a significant reduction in LTB4 (Figure 9B) compared to all other treatment groups at 3 and 5 days following infection. Ciprofloxacin also reduced LTB4 compared to PBS and Tβ4 alone, but remained significantly higher than the adjunct therapy. BLT1 mRNA expression (Figure 9D) was significantly reduced in both ciprofloxacin- and Tβ4 + ciprofloxacin-treated mice at both time points as well.

## 4. Discussion

Microbial keratitis is one of the most common and destructive of corneal diseases in humans, ultimately culminating in blindness. However, even less severe cases of keratitis develop some level of corneal opacity and compromised visual acuity. Development of corneal opacity is not limited to infection, though, and can be caused by injury and other eye diseases as well. Edema, inflammatory cell infiltration, fibrosis, and vascularization can all contribute to corneal opacity. Development of a therapeutic agent that clears or limits corneal scarring and promotes corneal wound healing without adverse side effects continues to be a major hurdle in the clinical setting and is the focus of the current study. Herein, we examined the therapeutic potential of Tβ4 as a combination therapy with antibiotics, revealing strong clinical implications relevant to human treatment of bacterial keratitis and potentially other conditions that result in corneal opacity.

Using an established murine model of bacterial keratitis, we examined the therapeutic potential of Tβ4 as an adjunct therapy to ciprofloxacin. Although it is best known for its wound-healing capacity, Tβ4 has anti-inflammatory properties [33,34] and is described as a multifunctional “moonlighting” protein [35]. Tβ4 treatment has been shown to modulate proinflammatory mediators in the cornea, as demonstrated in serious wound and burn models [12,34]. However, the exact mechanisms whereby Tβ4 exerts its anti-inflammatory effects are only superficially understood. To this end, we provide evidence suggesting that Tβ4 may carry out its profound effects, in part, through specialized proresolving mediator (SPM) pathway activation. We have previously demonstrated the importance of 15-LOX pathways in the pathogenesis of ocular infectious disease and that a well-balanced axis of 5-LOX/12-LOX/15-LOX pathways are essential in inhibiting inflammation and promoting resolution and tissue restoration [29]. Regulating these key proresolving circuits is a critical element of restoring ocular immune homeostasis. Results indicate that Tβ4 influences ‘resolution machinery’—including SPM enzymes (as observed with Tβ4 adjunctive therapy), receptors, and end products. A possible mechanism by which Tβ4 + ciprofloxacin is improving disease outcome compared to antibiotic alone may be through the activation of proresolving autacoid circuits. Though SPMs have been previously implicated in corneal wound healing [36], this is the first report indicating a potential, novel regulatory role for Tβ4 regarding these powerful pathways. 

We also provide evidence that proper bacterial clearance is crucial to the anti-inflammatory and wound-healing effects carried out by Tβ4. Previous in vitro work has shown that Tβ4 exhibits moderate antimicrobial activity against *P. aeruginosa* [18]; however, the in vivo model indicated otherwise. Further, as observed in the Tβ4-only-treated animals, the persistence of bacteria appears to impede the ability of this molecule to shift cytokine production from pro- to anti-inflammatory and prevent Tβ4-induced activation of tissue restoration pathways critical for corneal wound healing, whereas the adjunct treatment indicates potential synergistic bactericidal effects between Tβ4 and ciprofloxacin. In light of these findings, it also suggests a regulatory mechanism of Tβ4 activity that requires removal of the infectious agent and offers an avenue of further investigation. 

**Figure 10:**
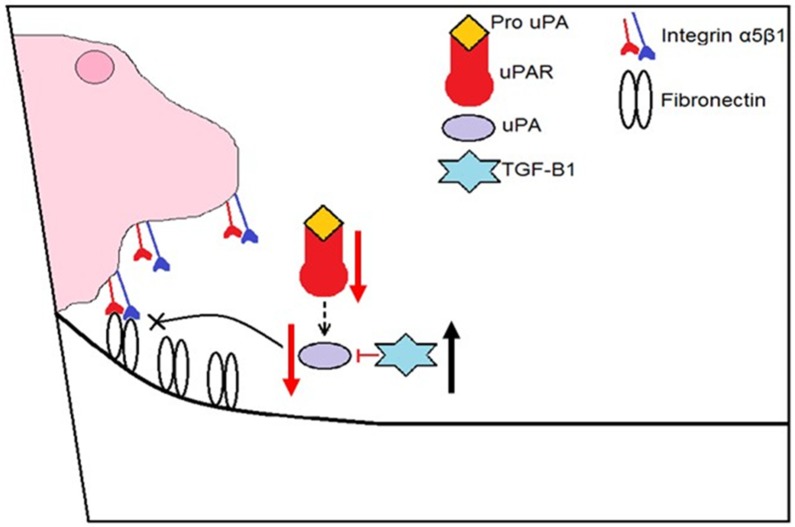
Schematic summarizing the effects of Tβ4 + ciprofloxacin on fibronectin, ITGα5β1, uPA, uPAR, and TGF-β1 during wound healing leading to successful migration of corneal epithelial cells (CEC) across the extracellular matrix (ECM).

To develop the most effective treatment possible for corneal infectious diseases, we must focus on two major aspects of pathogenesis—clearance of the invading pathogen and tissue restoration/homeostasis. As mentioned, previous studies have shown that Tβ4 promotes corneal wound healing. Sosne et al. have shown improved wound healing with Tβ4 treatment using dry eye and alkali burn models [33,34]. It has also been reported that Tβ4 promotes laminin-332 synthesis [12], a migration factor for corneal epithelial cells. Although the exact mechanism is not known, Tβ4 has been shown to stabilize HIF-1, known to bind the promotor region of the laminin-332 α3 chain [37,38].We have expanded upon this work to reveal that Tβ4 influences multiple wound-healing pathways, including fibronectin:integrin and uPA:uPAR. During a healthy wound-healing response, the former interaction signals epithelial cells to migrate [39]. As the wound heals, fibronectin and ITGα5β1 expression is known to decrease correlating with what we observed with the adjunctive therapy. Active uPA triggers the degradation of fibronectin, allowing the epithelial cells to move along the extracellular matrix. However, sustained uPA upregulation leads to excessive stromal degradation and corneal inflammation [40], which was observed in PBS controls and even Tβ4-only treatment. IL-1 has also been shown to promote collagen breakdown by keratocytes [40]. Sustained activation of uPA and elevated IL-1β levels in these treatment groups prevents proper migration of epithelial cells and further exacerbates the inflammatory response. TGF-β1 regulates uPA protease activity to restrict excessive degradation of the basement membrane. Tβ4 + ciprofloxacin treatment demonstrates a better balanced combination of uPA and uPAR (and is decreased compared to ciprofloxacin alone) along with increased TGF-β1 during the course of infection. Collectively, these findings suggest that Tβ4 promotes multiple aspects of this intricate cascade of events leading to successful corneal wound healing as illustrated in Figure 10.

Additionally, our findings indicate that Tβ4 adjunct therapy holds promise in overcoming limitations of current treatment modalities, namely the immunosuppressive effects associated with steroids. More importantly, we show that Tβ4 works synergistically with ciprofloxacin to enhance therapeutic action—a highly relevant aspect in light of increasing multidrug resistance in the major pathogens known to cause keratitis [1,2]. Taken together, these results strongly support further development of Tβ4 adjunct therapy, as it addresses critical aspects of corneal resolution and bacterial burden that together significantly improve disease outcome.

## Figures and Tables

**Table 1:** Nucleotide sequence of the specific primers used for PCR amplification.

Gene	Nucleotide Sequence	Primer
*b-actin*	5′- GAT TAC TGC TCT GGC TCC TAG C -3′	F
	5′- GAC TCA TCG TAC TCC TGC TTG C -3′	R
*TNF-a*	5′- ACC CTC ACA CTC AGA TCA TCT T -3′	F
	5′- GGT TGT CTT TGA GAT CCA TGC -3′	R
*IL-1b*	5′- CGC AGC AGC ACA TCA ACA AGA GC -3′	F
	5′- TGT CCT CAT CCT GGA AGG TCC ACG -3′	R
*iNOS*	5′- ACA GGA GAA GGG GAC GAA CT -3′	F
	5′- TGT TGC ATT GGA AGT GAA GC -3′	R
*CXCL2/MIP-2*	5′- TGT CAA TGC CTG AAG ACC CTG CC -3′	F
	5′- AAC TTT TTG ACC GCC CTT GAG AGT GG -3′	R
*LAMA3*	5′- GAC CTA CGT TCC ATC CTC CA -3′	F
	5′- CTG GCT TTT GTC CAT CTG CT -3′	R
*LAMB3*	5′- TTG ATT GAG CGC TCT TCT GA -3′	F
	5′- ATG CAG GGA TAG CTG ATG CT -3′	R
*LAMC2*	5′- TCC CCA GCT GAG TTA TTT CG -3′	F
	5′- CTG GCA GAA TTG TCC CTT GT -3′	R
*Fibronectin*	5′- GAA GGT TTG CAA CCC ACT GT -3′	F
	5′- CAT CCT CAG GGC TCG AGT AG -3′	R
*ITG* α*5*	5′- TGG ACC AAG ACG GCT ACA AT -3′	F
	5′- ATT GCC ATC CAG ATC TCG TC -3′	R
*ITG* β*1*	5′- GGG CAC ACT GTC TGG AAA CT -3′	F
	5′- TCG TCC ATT TTC TCC TGT CC -3′	R
*uPA*	5′- GCC TGC TGT CCT TCA GAA AC -3′	F
	5′- AAG AGA GCA GTC ATG CAC CA -3′	R
*uPAR*	5′- TTT GGA CCA GAG CTG TGA GA -3′	F
	5′- CAC CAT TGC AGT GGG TGT AG -3′	R
*TGF-* β*1*	5′- TCT CTG CTC TCT GCT GCT GAT ATG C -3′	F
	5′- AGG ACA AAT GGC TCT GAC ACA GTA CC -3′	R
*COX-2*	5′- TGA GCA ACT ATT CCA AAC CAG C -3′	F
	5′- GCA CGT AGT CTT CGA TCA CTA TC -3′	R
*5-LOX*	5′- ACT ACA TCT ACC TCA GCC TCA TT -3′	F
	5′- GGT GAC ATC GTA GGA GTC CAC -3′	R
*12-LOX*	5′- TAC CCT CCT GAG AAG CTG GA -3′	F
	5′- TCA TCT TCC TGC CAA CAC TG -3′	R
*12/15-LOX*	5′- GCG ACG CTG CCC AAT CCT AAT C -3′	F
	5′- ATA TGG CCA CGC TGT TTT CTA CC -3′	R
*FPR2*	5′- CCT TGG ACC GCT GTA TTT GT -3′	F
	5′- CCC CAG GAT ACA AAG CTC AA -3′	R
*BLT1*	5′- GCA TGT ATG CCA GTG TCC TG -3′	F
	5′- AAA AGA CAC CAC CCA GAT GC -3′	R

## Supplementary Materials

Supplementary File 1
