# Supplementary material for: Thymosin Beta-4 and Ciprofloxacin Adjunctive Therapy Improves Pseudomonas aeruginosa-Induced Keratitis
Source: Cells. 2018 Sep 20;7(10):145. doi: 10.3390/cells7100145 (PMC6210523; doi:10.3390/cells7100145)
Supplement: Supplementary File 1 [file cells-07-00145-s001.pdf]

Figure S1.

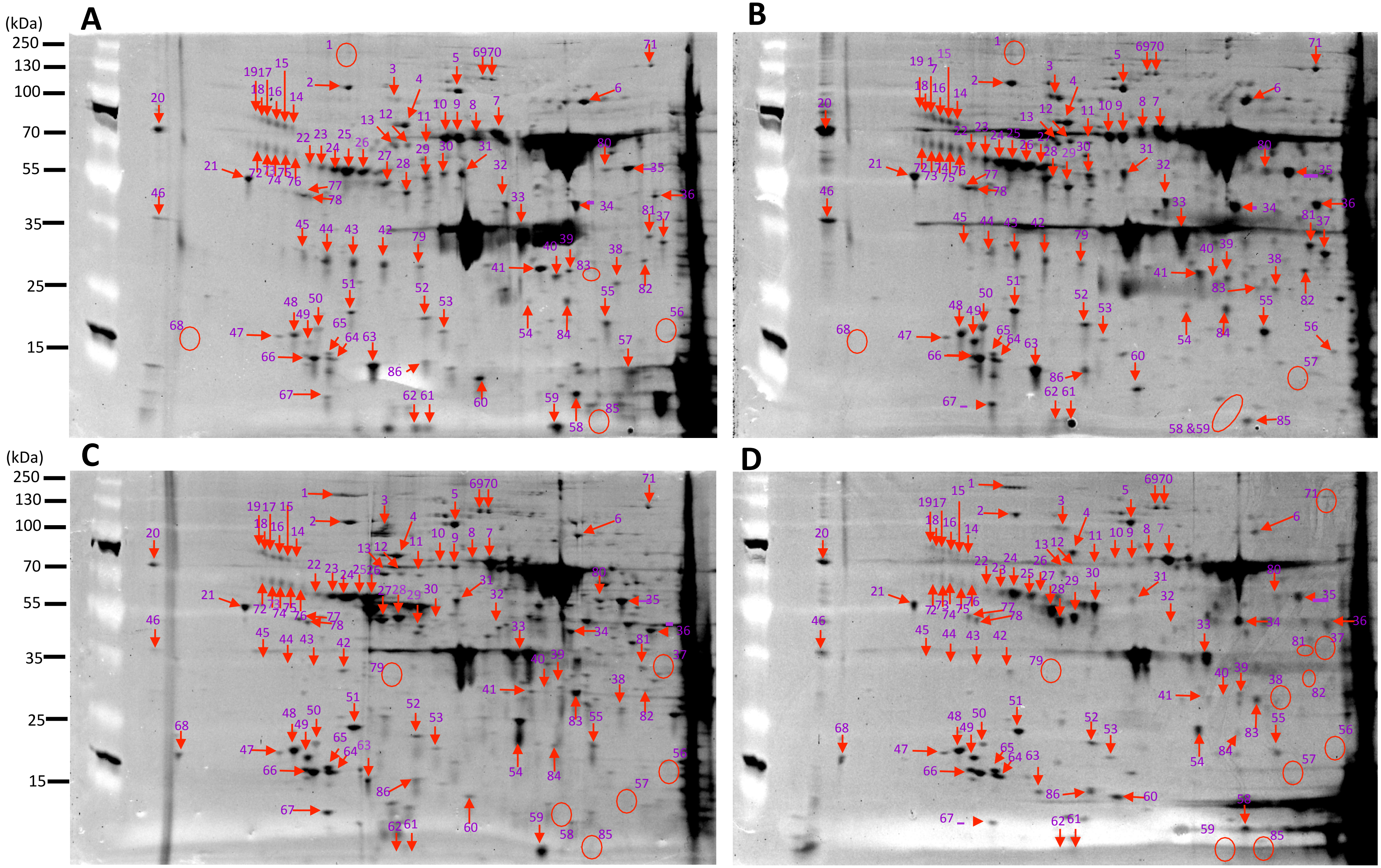

Figure S2.

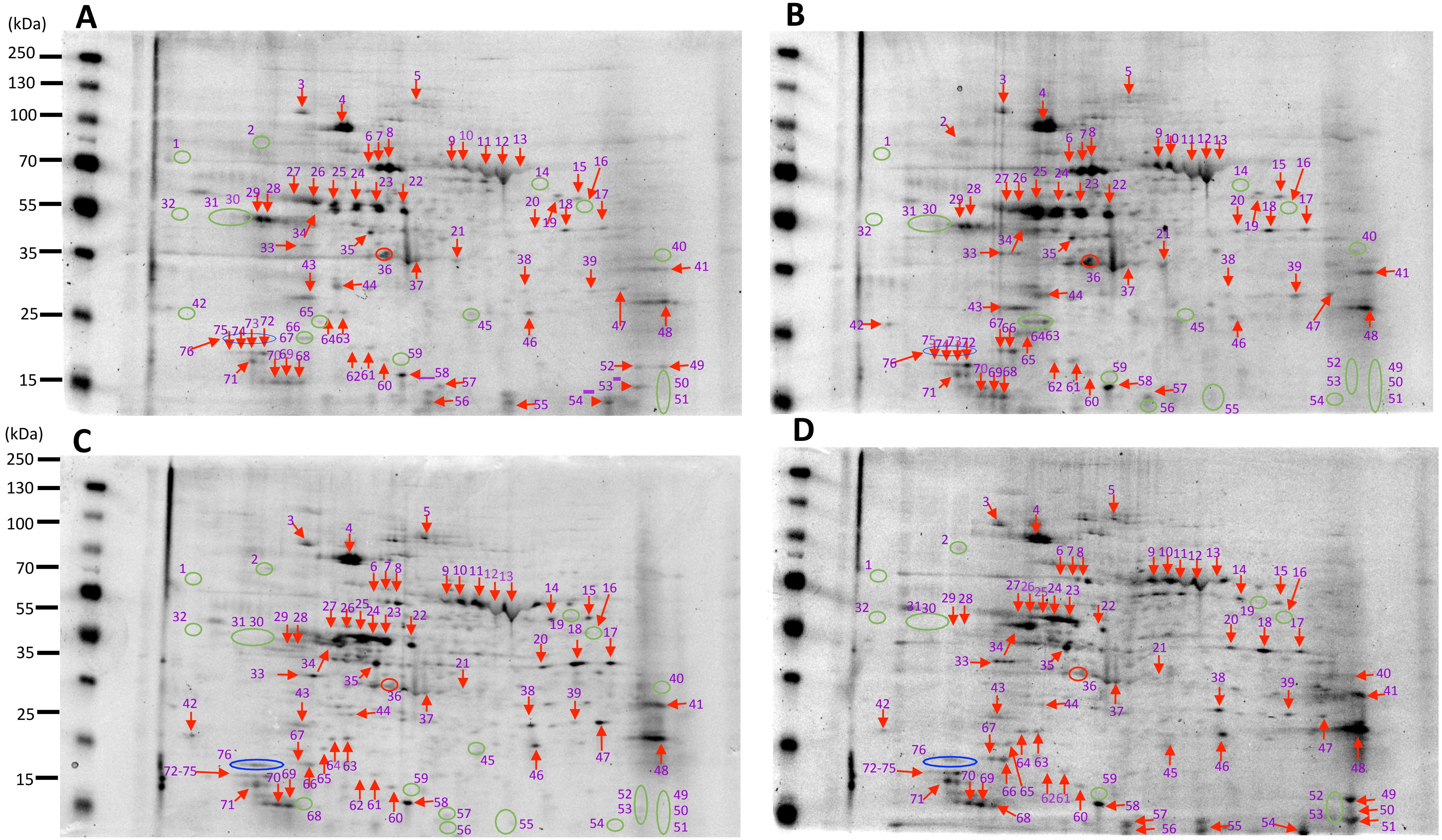

	3 days p.i.				5 days p.i.			
Spot #	PBS	Tβ4	Cipro	Tβ4+Cipro	PBS	Tβ4	Cipro	Tβ4+Cipro
1	-	-	+	++	-	-	+	+
2	+	++	++	++	++	+++	+++	+++
3	-	+	++	-	-	++	+++	-
4	+	+	+	+	++	++	++	++
5	+	++	+++	+++	+++	+++	+++	+++
6	++	++	+	+	++	+++	+	+
7	+++	+++	+++	+	+++	+++	+++	+++
8	+	++	+	+	+++	+++	+	+
9	+++	+++	++	++	+++	+++	++	-
10	+++	+++	++	++	+++	+++	++	-
11	+	+	+	+	+	++	+	+
12	+	++	+	+	+	+	+	-
13	+	++	++	++	+	+	++	+
14	+	+	-	-	+	+	+	+
15	+	+	+	+	+	++	+	+
16	+	+	+	+	+	++	+	+
17	+	+	+	+	+	++	+	+
18	+	+	+	+	+	++	+	+
19	+	+	-	-	+	+	+	+
20	++	+++	+	++	++	+++	+	++
21	+	++	++	++	++	++	++	++
22	+	+	-	-	+	+	+	+
23	++	++	+	+	+	+++	+	+
24	++	+++	++	++	+++	+++	+++	+++
25	+++	+++	+++	+++	+++	+++	+++	+++
26	+++	+++	+++	+++	+++	+++	+++	+
27	++	+++	+++	+++	++	++	+++	+++
28	+	+	+	++	++	++	+++	+++
29	+	+	+	++	++	++	+++	+++
30	+	+++	+++	+++	++	++	+	+++
31	+	+++	++	++	+++	+++	++	+
32	+	+	+	+	++	++	++	+
33	+++	+++	++	++	+++	+++	++	++
34	++	++	+	+	++	+++	+	++
35	++	++	+	++	++	+++	++	++
36	+	+	+	+	+	++	++	+
37	+	-	-	-	++	+++	-	-
38	+	+	-	-	+	+	+	-
39	+	+	+	-	++	++	+	+
40	+	+	-	-	++	+	-	-
41	++	++	+	+	+++	++	+	+
42	+++	+++	+	+	+++	+++	+	+
43	+++	+++	-	-	+++	+++	-	-
44	+++	+++	-	-	+++	++	-	-
45	++	+	-	-	+	+	-	-
46	+	++	-	+	+	+++	-	+
47	+	+	+	+	+	+	+	+
48	+	+	+	++	++	+++	+++	+++
49	+	+	++	++	+	+++	+++	+++
50	+	+	+	+	+	++	+	+
51	+	+	++	++	+	+	++	++
52	+	+	+	+	+	++	+	+
53	+	+	+	+	+	+	+	+
54	+	++	++	++	+	++	+++	++
55	+	+	-	-	++	+++	+	+
56	++	-	-	-	-	+	-	-
57	+	-	-	-	++	-	-	-
58	+	-	-	-	++	-	-	++
59	++	-	-	-	+++	-	+++	-
60	++	++	+	+	++	++	+	++
61	-	-	+	+	+	-	-	-
62	-	-	+	+	+	-	-	-
63	+++	+++	+	+	+++	+++	+	+
64	+	+	+	++	+	+++	+++	+++
65	+	+	+	++	+	+++	+++	+++
66	++	++	++	++	++	++	++	++
67	-	-	-	+	+	++	++	+
68	-	-	+	++	-	-	++	++
69	+	+	++	++	++	++	++	++
70	+	+	++	++	++	+=	++	++
71	-	-	-	-	+	+	+	-
72	+	+	-	-	+	++	+	+
73	+	+	+	+	+	++	+	+
74	+	+	+	+	+	++	+	+
75	+	+	+	+	+	++	+	+
76	+	+	+	+	-	++	-	-
77	+	+	+	+	+	+	++	++
78	+	+	+	+	+	+	++	++
79	+	++	-	-	+	+	-	-
80	-	-	-	-	+	++	++	+
81	+	-	-	-	+	+++	+	-
82	+	-	-	-	+	++	-	-
83	-	-	-	-	-	-	+++	++
84	+	+	-	-	++	+++	+	+
85	-	-	-	-	-	+++	-	-
86	+	+	+	+	+	++	+	++

Table S1. Qualitative comparison of total protein expression between treatment groups at 3 and 5 days p.i.
Key: -, No Expression; +, Low Expression; ++, Medium Expression; +++, High Expression

	3 days p.i.				5 days p.i.			
Spot #	PBS	Tβ4	Cipro	Tβ4+Cipro	PBS	Tβ4	Cipro	Tβ4+Cipro
1	-	-	-	+	-	-	-	-
2	-	+	+	-	-	+	-	-
3	+	+	+	+	+	+	+	+
4	+++	+++	+++	+++	+++	+++	+++	+++
5	-	-	+	+	-	-	+	+
6	+	+	-	-	+	+	-	-
7	+++	+++	-	-	+++	+++	+	+
8	+++	+++	+	+	+++	+++	+	+
9	+	+	+	++	+	++	++	++
10	+	+	+	++	+	++	++	++
11	++	++	+	++	+	+	+	+
12	++	+++	++	++	++	++	++	++
13	-	+	+	++	-	-	++	++
14	-	+	+	++	-	-	+	+
15	++	-	-	++	+	+	+	++
16	-	-	+	+	-	-	-	-
17	+	+	++	+	+	+	++	+
18	++	++	+++	+++	++	++	+++	+++
19	+	+	-	+	+	+	-	-
20	+	+	+	+	-	+	+	+
21	+	+	-	-	+	+	-	-
22	++	++	++	++	++	+++	++	+
23	+++	+++	+	+	+++	+++	+++	++
24	+++	+++	+	+	+++	+++	+++	++
25	+++	+++	+	+	+++	+++	+++	++
26	+++	+++	+	+	+++	+++	+++	++
27	+++	+++	-	+++	+	+	+	+
28	+	++	-	-	++	++	-	-
29	++	++	-	-	++	++	-	-
30	+	+	-	-	-	-	-	-
31	+	+	-	-	-	-	-	-
32	-	-	-	+	-	-	-	-
33	-	+	+	+	-	-	+	+
34	+++	+	-	-	-	-	-	-
35	+	++	++	++	+	+	++	++
36	++	+	-	-	+++	+++	+	+
37	+	+	+	+	+	+	+	+
38	-	-	-	+	-	-	+	+
39	+	+	+	+	+	+	++	+
40	-	+	-	-	-	-	-	-
41	+	++	++	++	-	++	++	+++
42	+	+	+	++	-	-	-	-
43	-	-	-	-	-	+	-	-
44	-	-	-	-	+	+	-	-
45	-	+	+	+	-	-	-	-
46	+	+	+	++	+	+	++	++
47	+	+	+	+	+	+	-	+
48	++	++	++	++	+	++	++	+++
49	-	-	+	-	+	-	-	++
50	-	-	+	-	-	-	-	-
51	-	-	+	+	-	-	-	++
52	-	-	-	-	+	-	-	-
53	-	-	-	-	+	-	-	-
54	-	-	++	-	+	-	-	++
55	-	-	-	-	+	-	-	+
56	-	-	+	+	+	-	-	+
57	-	-	-	-	+	+	-	++
58	+++	++	+++	+++	++	+++	++	++
59	++	-	-	-	-	-	-	-
60	-	-	-	-	-	-	+	+
61	-	-	-	-	+	+	+	+
62	-	-	-	-	+	+	-	-
63	+	-	-	+	+	-	+	+
64	+	-	+	+	+	+	+	+
65	+	+	-	+	-	+	+	+
66	+	+	++	+	+	++	+	++
67	-	+	+	+	+	+	-	+
68	-	+	-	-	-	+	-	+
69	+	++	++	+	+	+	+	++
70	+	+	+	++	+	+	+	++
71	+	+	+	+	+	+	+	++
72	+	+	+	+	+	++	+	+
73	+	+	+	+	+	+	+	+
74	+	+	+	+	+	+	+	+
75	+	+	+	+	+	+	+	+
76	-	-	+	++	-	-	+	+

Table S2. Qualitative comparison of phosphprotein expression between treatment groups at 3 and 5 days p.i. Key: -, No Expression; +, Low Expression; ++, Medium Expression; +++, High Expression
